# Editorial: Recent advances in abiotic stress research for Asia

**DOI:** 10.3389/fpls.2024.1428049

**Published:** 2024-06-13

**Authors:** Dung Tien Le

**Affiliations:** VKTECH Research Center, Nguyen Tat Thanh University, Ho Chi Minh City, Vietnam

**Keywords:** abiotic stress, heat stress, drought, high salinity, anaerobic stress, Asia, crop sciences

Abiotic stress in crops poses a significant threat to global agriculture and is primarily caused by climate extremes and side effects of industrialization. Asia is particularly at risk, due to droughts and rising salinity, changes river flows, which is jeopardizing food production across the continent. Unlike other regions with large-scale, mechanized farms, Asia relies heavily on smallholder farmers who produce 90% of the world’s rice and a significant portion of other key crops like wheat, vegetables, and fruits.^1^

This Research Topic, which focuses on recent advances in abiotic stress in Asia, aims to showcase the research achievements from within the region and facilitate collaboration across the world. In addition, this Research Topic will look to promote research that helps to tackle the problems specific to smallholder farmers in Asia. We believe that promoting research and collaboration in this space will be beneficial not only for Asia but also for the rest of the world when it comes to addressing food security challenges. In particular, we aim to public studies that could contribute to a better understanding of plant adaptation to abiotic stress, establishing strategies for improving abiotic stress tolerance in plants, including transgenic, gene-editing and chemical priming approaches.

With the collaboration of Professor Weiqiang Li of Henan University, China, the Research Topic received 14 eligible manuscripts, of which, seven were accepted, after rigorous reviews. The published articles covered all major abiotic stresses, including acidic soil, osmotic stress, anaerobic conditions, heat, heavy metal pollution and water logging stresses. The contributing authors came from 27 institutions spanning 6 countries in Asia, including China, India, Indonesia, Japan, Malaysia and Vietnam. Notably, one was a review on the function of STOP1 in adaptation to acidic soil from the newly established Hainan Yazhou Bay Seed Laboratory, which was envisioned to be a “Silicon Valley” for seed technology^2^ in China, is featured. Contributions from Japan were mostly from the major well-known mega institutions like Japan International Research Center for Agricultural Sciences (JIRCAS), RIKEN, the National Institute of Agrobiological Science (NIAS), the University of Tokyo, Tokyo University of Agriculture, Hokkaido University and the National Institute of Advanced Industrial Science and Technology (AIST). Contributions from India were also originating from highly reputed institutions under the Indian Council of Agricultural Research (ICAR). Here, I provided brief summaries of the articles published in this topic.

The rapidly growing world population has higher food requirements which need to be met through better yield and, in certain situations, utilization of less favorable arable lands. Together with increasing industrialization, being able to produce crops under acidic soil will help. In this topic, Li and Tian have reviewed and highlighted a recent understanding about the transcription factor (TF) family called STOP1 (Sensitive to proton rhizotoxicity 1) (Li and Tian), a versatile TF involving in regulation of various nutrient uptake in plant under abiotic stress, in helping plants coping with acidic soil.

Transcriptional responses are one of the key steps in plant’s strategies to adapt to stress. Work by Lim et al., from the Forestry Division of JIRCAS (Lim et al.), reported transcriptional responses in oil palm during a 2-year period of seasonal variations and precipitation. The authors reported a significant response to waterlogging in the stem core but not in the leaf samples, including the details of 19 genes encoding TFs. Their report will provide guidance for further investigation.

Phytoremediation of heavy metal contaminated soil represents an opportunity to not only to improve the quality of the agricultural products but also possesses potential to enable the cultivation in low quality soil. This adds extra arable land area for either food or biomass production. To this end, our Research Topic published an interesting research article by Zhang et al. from Fujian, China. In their report, the authors proposed and tested the application of the ectomycorrhizal fungi (ECMF), *Cenococcum geophilum* (*C. geophilum*) on the adaptation of Chinese red pine, *Pinus massoniana (P. massoniana)* to metal polluted soil (Zhang et al.). They found that the ecotypes of the fungi that are low or non-tolerant to heavy metal promoted the accumulation of the heavy metals in the pine plants, and the tolerance of the pine plants against heavy metals might have been achieved via the enhancement of the antioxidant system as evidence by the increase in their transcriptions.

The topic of plant immunity has been of interest to plant research communities as well. Scientists are especially interested in how plants could be primed by a mild stress and then become tolerant to the subsequent severe stress. This area of focus received attention and we had the privilege to publish three research articles from authors in Japan. The research from Tokyo University of Agriculture (TUAT) reported the identification of a gene encoding a protein of the nuclear pore complex, NUCLEOPORIN 85 (NUP85), which when mutated could promote osmotolerance in *Arabidopsis* via suppressing the nuclear translocation of a nucleotide-binding leucine-rich repeat (NLR) protein call ACQOS (Mori et al.). In another case, a report from Seki’s group of RIKEN Center for Sustainable Resource Science (RIKEN CSRS) elucidated a benefit on vegetative growth and yield of tomato (*Solanum lycopersicon* L) under heat stress when primed by a treatment with a low concentration of ethanol (Todaka et al.). Previously, this group also reported the effects of ethanol priming on high salinity tolerance in *Arabidopsis* (Nguyen et al., 2017) and drought stress avoidance in cassava (Vu et al., 2022). Researchers from Miyagi University also reported the role of heat stress memory in regulating the expression of nitrogen transporter genes in the filamentous red alga ‘Bangia’ sp. ESS1 (Sato et al.).

Finally, a perspective article from a group of researchers from India, Indonesia and Malaysia reported the preliminary result on the feasibility to use Autofluorescence−spectral imaging (ASI) as a method to differentiate the soybean seeds that have variations in their tolerance to pre-germination anaerobic stress (Rajendran et al.). This method, once verified, can be helpful to accelerate breeding for selection of stress-tolerant phenotypes, and may contribute to a method for quick assessment of seed vigor, a routine step in seed quality assurance.

As a conclusion, what we have published on the current topic is important but may not represent all on-going efforts to address the challenges for agriculture in Asia. To contribute to a food-secure world, plant research needs to continue focusing on developing climate-resilient crops/varieties, including those that enable innovative agronomic practices, such as precision agriculture, automation, and chemical priming. I believe regional cooperation is crucial to facilitate solving challenges in food production across Asia., by knowledge and resource sharing.

## Author contributions

DL: Conceptualization, Data curation, Formal analysis, Funding acquisition, Investigation, Methodology, Project administration, Resources, Software, Supervision, Validation, Visualization, Writing – original draft, Writing – review & editing.

## Footnotes

1 FAO, https://www.fao.org/markets-and-trade/commodities/rice/en/ (accessed date May 5^th^, 2024).

2
http://en.people.cn/n3/2022/0414/c90000-10083809.html (accessed date May 5^th^, 2024).
